# Mecanismos do tromboembolismo venoso no câncer: uma revisão da literatura

**DOI:** 10.1590/1677-5449.007817

**Published:** 2017

**Authors:** Marcos José Pereira Renni, Mônica Hermida Cerqueira, Ingrid de Araújo Trugilho, Mario Lúcio Cordeiro Araujo, Marcos Arêas Marques, Hilton Augusto Koch

**Affiliations:** 1 Instituto Nacional do Câncer – INCA, Divisão de Pesquisa Clínica, Rio de Janeiro, RJ, Brasil.; 2 Instituto de Hematologia Arthur de Siqueira Cavalcanti – HEMORIO, Rio de Janeiro, RJ, Brasil.; 3 Universidade do Estado do Rio de Janeiro – UERJ, Unidade Docente Assistencial de Angiologia, Rio de Janeiro, RJ, Brasil.; 4 Universidade Federal do Rio de Janeiro – UFRJ, Hospital Universitário Clementino Fraga Filho, Departmento de Radiologia, Rio de Janeiro, RJ, Brasil.

**Keywords:** trombose venosa, câncer, fatores de risco

## Abstract

Existe uma estreita relação entre o tromboembolismo venoso e o câncer. Pacientes com neoplasias apresentam maior incidência de eventos tromboembólicos em sua evolução clínica. A ocorrência desses eventos é considerada um marcador preditivo negativo nesse grupo de pacientes. Revisamos, então, a ativação dos mecanismos de coagulação neste grupo de pacientes. Trata-se de um processo complexo e multifatorial, relacionado tanto a características tumorais, estadiamento clínico, agressividade da doença e sítios tumorais, dentre outros. Novos biomarcadores vêm sendo pesquisados ao longo dos anos na tentativa de correlacioná-los ao risco trombótico, visando uma intervenção que melhore a evolução clínica desses pacientes oncológicos.

## INTRODUÇÃO

O câncer e seus diversos tratamentos são reconhecidos como fatores de risco independentes para o desenvolvimento de tromboembolismo venoso (TEV)^1^^,^ 2. A associação clínica entre neoplasias e hipercoagulabilidade é conhecida há mais de um século, e os eventos tromboembólicos são mais frequentes em pacientes oncológicos – um em cada cinco pacientes com neoplasia apresentará TEV durante a evolução natural da doença^3^.

O TEV inclui um espectro de quadros clínicos que vai desde trombose venosa profunda (TVP) e superficial até embolia pulmonar (EP)^4^^,^ 5. É a segunda causa de morte em pacientes com neoplasias, entre os quais um em cada sete tem o óbito relacionado a complicações, especialmente durante o período de internação hospitalar. Desses pacientes, 60% têm câncer em sítio único ou doença metastática limitada. Segundo Prandoni et al., eles podem sobreviver por mais tempo quando não apresentam TVP ou EP^6^.

Os tipos de câncer mais prevalentes entre pacientes com TEV são os de mama, colorretal e de pulmão, o que reflete a prevalência dessas neoplasias na população em geral^2^^,^ 7 ^-^ 9. Embora os tumores sólidos tenham sido historicamente mais associados ao TEV, dados mais recentes sugerem risco similar em pacientes com neoplasias de origem hematológica^10^^-^ 12.

Há vários mecanismos que se sobrepõem e interagem e podem explicar o aumento da incidência de TEV nos pacientes com neoplasias. Trousseau, em 1865, observou que alguns pacientes apresentavam eventos trombóticos inesperados, incomuns, com padrão migratório, e, posteriormente, manifestavam uma malignidade visceral^13^^,^ 14. A síndrome de Trousseau, como é conhecida, pode ser descrita de muitas formas, incluindo TEV espontâneo em associação com doença neoplásica oculta, que pode ser, em alguns casos, a primeira manifestação da doença neoplásica^4^^,^ 5 ^,^ 15.

Essa síndrome tem sido amplamente utilizada para englobar todos os aspectos do TEV associados ao câncer. Entretanto, o risco de TEV não é igual para todos os pacientes com câncer ou para o mesmo paciente ao longo do tempo^1^^,^ 12 ^,^ 16. Determinadas situações como características tumorais, localização anatômica, grau de agressividade e condições clínicas do paciente facilitam ou não o aparecimento do TEV em um determinado momento da evolução da doença^4^^,^ 17 ^,^ 18.

## EPIDEMIOLOGIA

Diversos estudos estão sendo realizados para definir com mais exatidão a prevalência de TEV associado ao câncer, pois se acredita que essa associação ainda esteja subestimada. Estudos evidenciaram que muitos pacientes que desenvolveram TEV foram diagnosticados com algum tipo de câncer nos 12 meses subsequentes ao surgimento do evento tromboembólico^2^^,^ 3 ^,^ 9. A magnitude dessa complicação é tamanha que se estima que pacientes oncológicos que desenvolvem TEV apresentem 94% de probabilidade de morte nos seis meses seguintes ao episódio. Portanto, o TEV pode ser considerado um marcador preditivo negativo na sobrevida dos pacientes oncológicos^18^^-^ 21.

O câncer por si só está associado a um risco quatro vezes maior de desenvolvimento do TEV, enquanto a quimioterapia aumenta esse risco em seis vezes. Os pacientes em terapia com citotóxicos são responsáveis por 13% dos episódios de TEV na população oncológica^6^^,^ 8 ^,^ 22 ^,^ 23. Um estudo de coorte retrospectivo descrito por Blom et al., em 2005, relata que os pacientes em quimioterapia tiveram 2,2 vezes mais risco de desenvolvimento de TEV^1^^,^ 11 ^,^ 24. A incidência de TEV nos pacientes oncológicos em pós-operatório é duas vezes maior quando comparada aos pacientes em pós-operatório, porém livres de neoplasias^6^^,^ 9 ^,^ 10. Fatores como imobilização prolongada e implante de cateteres venosos centrais também aumentam o risco de TEV nesse grupo^10^^,^ 25. Portanto, a estimativa aproximada da incidência anual de TEV em uma população com neoplasia pode ser de aproximadamente 1/200 pacientes^26^. Porém, a incidência de TEV nos pacientes com diferentes tipos de neoplasias permanece, em grande parte, desconhecida devido à heterogeneidade da população e à dificuldade de realização de grandes estudos epidemiológicos.

Os pacientes oncológicos também apresentam um risco elevado de recorrência de TEV, particularmente nos meses subsequentes à interrupção do tratamento anticoagulante. Esse risco chega a ser de duas a 3,5 vezes maior na comparação com pacientes que desenvolveram TEV não relacionado a neoplasias^15^^,^ 21 ^,^ 27. No seguimento de pacientes, foi observado que o TEV recorrente pode ocorrer mesmo na vigência de anticoagulação plena, o que sugere uma doença mais agressiva, com um pior prognóstico^1^^,^ 6 ^,^ 27.

## FATORES DE RISCO E MECANISMO PATOGÊNICO PARA A FORMAÇÃO DE TROMBOS

A avaliação do risco para desenvolver TEV é um processo dinâmico e envolve uma série de fatores como idade avançada, gênero, etnia (maior em afroamericanos e menor em asiáticos), sítios tumorais (cérebro, pâncreas, estômago, pulmão, bexiga, tumores ginecológicos e hematológicos), estágio da doença e período inicial após o diagnóstico^15^^,^ 28 ^-^ 30. Há ainda os fatores relacionados ao tratamento, como cirurgias, hospitalização, quimioterapia, terapias antiangiogênicas, agentes estimulantes da eritropoiese e contagem elevada de plaquetas pré-quimioterapia^10^^,^ 12 ^,^ 31 ^,^ 32.

As propriedades pró-trombóticas, específicas de cada tumor, contribuem para o processo de crescimento tumoral e sua disseminação. As células neoplásicas podem ativar o mecanismo de coagulação através de várias substâncias, como as pró-coagulantes, inibidoras da fibrinólise, citocinas, cisteína protease, pró-inflamatórias e pró-angiogênicas, e por interação direta com endotélio vascular, leucócitos e plaquetas^33^^-^ 36.

A formação de trombina, enzima efetora final dos mecanismos de coagulação, e a produção de fibrina, produto final da ativação da coagulação sanguínea, são dependentes dos mecanismos de progressão tumoral. Além disso, propriedades tumorais pró-trombóticas podem interferir na malignidade através de mecanismos independentes de coagulação. Serão detalhados a seguir alguns dos principais mediadores e mecanismos para desenvolvimento de TEV nas neoplasias.

## TRÍADE DE VIRCHOW

Os três elementos-chave da tríade de Virchow, relatados em 1856 por Rudolf Virchow e atualmente descritos como estase venosa, lesão endotelial e hipercoagulabilidade, acrescidos das informações atuais sobre os elementos do sangue e suas complexas interações no processo fisiopatológico da trombogênese, são ferramentas úteis para explicar a etiopatogenia do TEV nos pacientes com neoplasia^37^. Os pacientes oncológicos podem apresentar alterações no endotélio vascular acarretadas pela doença ou ainda secundárias aos tratamentos aos quais são submetidos. Além disso, estão sujeitos a imobilizações prolongadas pelo curso da doença ao longo do tratamento e a alterações hematológicas decorrentes da atividade tumoral.

**Estase venosa:** o repouso prolongado no leito e a compressão extrínseca dos vasos sanguíneos por massas tumorais podem acarretar estase venosa.

**Lesão endotelial:** é secundária a vários fatores, que podem atuar de forma local, como a invasão direta da veia pelo tumor ou pelo implante de cateter venoso central, ou a distância, como a lesão endotelial secundária ao tratamento quimioterápico^38^. O endotélio produz substâncias como o óxido nítrico e a prostaciclina, que são vasodilatadoras e mantêm as plaquetas no estado não ativado, evitando a sua agregação. Porém, quando a camada endotelial é rompida, as plaquetas são expostas a ligações subendoteliais, para as quais possuem receptores específicos que iniciam o seu processo de ativação^38^. O processo inflamatório, através da liberação de mediadores, estimula o endotélio a produzir o fator tecidual (FT), inibidor do ativador do plasminogênio do tipo I (PAI-1), e a reduzir a síntese da trombomodulina, diminuindo, dessa forma, a sua capacidade protetora^39^. Esse mecanismo favorece a produção de fibrina através de uma regulação crescente do FT e da produção de micropartículas. Os pacientes com neoplasia apresentam nível elevado de FT circulante^36^^,^ 39. Além do FT, os fatores pró-coagulantes nas neoplasias incluem a produção de substâncias trombogênicas como o fator coagulante e citocinas inflamatórias, bem como a interação de células tumorais com monócitos, macrófagos, plaquetas e células endoteliais^36^^,^ 39.

**Hipercoagulabilidade:** nos pacientes oncológicos, a hipercoagulabilidade é produzida por um conjunto complexo de mecanismos^36^:

- Lançamento de micropartículas derivadas do tumor, ricas em potentes fatores teciduais pró-coagulantes e citocinas capazes de provocar a ativação endotelial.
- Danos aos mecanismos de defesa das células endoteliais.
- Redução dos níveis plasmáticos dos inibidores naturais da coagulação: antitrombina e proteínas C e S.
- Aumento das interações adesivas entre as células tumorais, células do endotélio vascular, plaquetas e monócitos/macrófagos, mediado por interações de selectina^36^^,^ 37 ^,^ 40.

Presume-se que a ativação da coagulação nos pacientes oncológicos seja simplesmente uma reação do hospedeiro ao desenvolvimento do tumor. Assim, não desempenha um papel fundamental nos eventos moleculares que levam ao desenvolvimento do câncer^36^^,^ 40.

## SUBSTÂNCIAS PRÓ-COAGULANTES

As células tumorais produzem substâncias pró-coagulantes, como o FT, o fator de necrose tumoral (TNF) e o fator de crescimento endotelial vascular (VEGF), que estão envolvidas no crescimento da massa tumoral e na ativação do mecanismo de coagulação^35^. O FT é o ativador primário da coagulação em indivíduos saudáveis. Ele se expressa na superfície da maioria das células não vasculares e forma um complexo com o fator VII (FVII) para ativar os fatores IX (FIX) e X (FX) por proteólise.

O FT circula na forma de micropartículas em quantidade aumentada em várias doenças, entre elas o câncer. É interessante observar que o FT parece predizer a agressividade tumoral em humanos e tem sido correlacionado, embora retrospectivamente, com o aumento da angiogênese tumoral, da taxa de crescimento rápido, das metástases e, finalmente, da propensão a desenvolver TEV^36^.

Em células vasculares normais, a expressão do FT é rigidamente controlada. Entretanto, parece ocorrer um aumento da expressão do FT pelas células neoplásicas, induzido por estímulos inflamatórios, como as citocinas interleucina 1 e o TNF, bem como os lipopolissacarídeos de bactérias. Desse modo, o início da coagulação do sangue pelo FT pode acontecer diretamente através da sua expressão na superfície de células neoplásicas ou indiretamente através da sua ação em células endoteliais, monócitos, macrófagos e fibroblastos, após o estímulo inflamatório^4^^,^ 24.

A regulação da expressão de FT em células tumorais é controlada, em nível molecular, por vários oncogenes, como parece ocorrer para a ciclo-oxigenase 2 (COX-2), importante regulador da função plaquetária e do PAI-1, um inibidor da fibrinólise. Além disso, a ligação de receptores de protease-ativada (PARs) pelo FT, FVIIa, FXa e/ou trombina já demonstrou ser importante para angiogênese tumoral, crescimento e metástases^40^^-^ 43.

Quanto ao papel emergente da atividade não coagulante do FT, é relevante, em particular, a sua capacidade de modular a expressão do VEGF pelas células neoplásicas e células vasculares normais. Essa propriedade regula a neovascularização do tumor e fornece uma importante relação entre pacientes com câncer e ativação da coagulação, inflamação, trombose, crescimento tumoral e metástases^42^^,^ 44.

As proteínas de coagulação do sangue desempenham pelo menos dois papéis importantes na biologia tumoral: o papel pró-coagulante intravascular e extravascular, que leva ao depósito de fibrina; e o aprimoramento das células tumorais na angiogênese, crescimento e metástase. Essa dupla função ocorre para o FT e para o complexo de FVII e trombina, que se ligam e ativam os PARs em células tumorais, células endoteliais e plaquetas^40^^,^ 42. As manifestações trombóticas decorrentes estão intimamente relacionadas à biologia tumoral e tornam o paciente susceptível ao desenvolvimento de TEV devido ao estímulo ocasionado pelo crescimento tumoral e pelos mecanismos fisiopatológicos envolvidos na sua gênese.

Tem sido sugerido também que, em alguns pacientes com câncer, o tumor gere protease cisteínica, iniciando assim a coagulação do sangue, conforme apresentado no estudo de Gordon et al. (1981)^45^. Eles descreveram a protease cisteínica, que ativa diretamente o FX na ausência do FVII. O consenso atual é que essa protease pode desempenhar um papel importante no estado pró-trombótico de algumas neoplasias, mas ainda faltam dados para tal comprovação^36^^,^ 40.

## P-SELECTINA

A P-selectina é uma molécula de adesão que interage com plaquetas, células endoteliais e leucócitos. Ela aumenta a expressão do FT em células endoteliais e monócitos, e os seus níveis plasmáticos elevados têm sido associados a um aumento do risco de TEV em pacientes oncológicos. Esse dado pode ser utilizado para discriminar diferentes riscos de TEV^46^^,^ 47; entretanto, sua utilização como um marcador de risco ainda é limitada pela falta de disponibilidade do seu amplo espectro na prática clínica^3^^,^ 10 ^,^ 46 ^,^ 47.

## BIOMARCADORES PARA O RISCO DE TEV

Além do FT das células tumorais, FT circulante e P-selectina solúvel, citados anteriormente, existem ainda outros possíveis biomarcadores para o risco de TEV, como contagem elevada de plaquetas pré-quimioterapia, contagem elevada de leucócitos pré-quimioterapia, dímero D e proteína C reativa^10^^,^ 12 ^,^ 48.

A leucocitose (> 11.000/mm^3^) foi recentemente identificada como fator de risco independente para o TEV, associada ao aumento do risco nos pacientes oncológicos em início de tratamento com quimioterapia. Além disso, a taxa de TEV em pacientes que tiveram leucocitose persistente, após o primeiro ciclo de quimioterapia, foi significativamente superior à taxa daqueles com leucopenia. A leucocitose pode ser um marcador de maior agressividade do câncer, não utilizado pelos tradicionais indicadores de prognóstico como estágio da doença. A contagem elevada de plaquetas na pré-quimioterapia também foi identificada como um fator de risco para trombose associada ao câncer^16^. A dosagem da hemoglobina (< 10 g/dl-1) também é considerada um marcador biológico para o risco trombótico^10^^,^ 12 ^,^ 36 ^,^ 48.

A trombocitose foi definida como uma contagem de plaquetas igual ou superior a 350.000/mm^3^, e foi observada em 21,9% dos 4.405 pacientes analisados em um estudo prospectivo no início do tratamento quimioterápico ambulatorial. Esses pacientes apresentaram uma taxa três vezes maior de TEV. O risco elevado de desenvolver TEV, com a contagem de plaquetas elevada, persiste enquanto o paciente estiver em quimioterapia^24^.

## CONCLUSÃO

A ativação da coagulação sanguínea em pacientes com câncer é complexa e multifatorial, o que torna esses pacientes especialmente suscetíveis ao TEV. As células neoplásicas podem ativar o mecanismo de coagulação através de várias substâncias, como citocinas, cisteína protease, pró-coagulantes e pró-inflamatórias, além da sua interação direta com endotélio vascular, leucócitos e plaquetas. Os mecanismos pró-trombóticos estão relacionados ao paciente e ao tipo tumoral, que exerce uma ação específica no processo trombótico. Portanto, é fundamental o conhecimento desses mecanismos para a prevenção de TEV nesses pacientes. Por se tratar de um evento frequente e de impacto negativo na evolução clínica, ao identificarmos o subgrupo mais propenso a desenvolver TEV, poderemos intervir rapidamente quer seja na profilaxia ou no tratamento de forma mais eficaz nessa população, levando a menor morbidade e maior sobrevida.
