# Supplementary material for: Mecanismos do tromboembolismo venoso no câncer: uma revisão da literatura
Source: J Vasc Bras. 2017 Oct-Dec;16(4):308–13. [Article in Portuguese] doi: 10.1590/1677-5449.007817 (PMC5944308; doi:10.1590/1677-5449.007817)
Supplement: Translation

# Translation: Mechanisms of venous thromboembolism in cancer: a literature review

**Authors:** Marcos José Pereira Renni, Mônica Hermida Cerqueira, Ingrid de Araújo Trugilho, Mario Lúcio Cordeiro Araujo, Marcos Arêas Marques, Hilton Augusto Koch

**Affiliations:** 1 Instituto Nacional do Câncer – INCA, Divisão de Pesquisa Clínica, Rio de Janeiro, RJ, Brazil.; 2 Instituto de Hematologia Arthur de Siqueira Cavalcanti – HEMORIO, Rio de Janeiro, RJ, Brazil.; 3 Universidade do Estado do Rio de Janeiro – UERJ, Unidade Docente Assistencial de Angiologia, Rio de Janeiro, RJ, Brazil.; 4 Universidade Federal do Rio de Janeiro – UFRJ, Hospital Universitário Clementino Fraga Filho, Departmento de Radiologia, Rio de Janeiro, RJ, Brazil.

**Keywords:** venous thrombosis, cancer, risk factors

## Abstract

There is a strong relationship between venous thromboembolism and cancer. Patients with tumors have a higher incidence of thromboembolic events in their clinical evolution. The occurrence of such events is considered a negative predictive marker in this group of patients. Thus, we aim to review activation of coagulation mechanisms in this group of patients. Activation of coagulation mechanisms in cancer patients is a complex and multifactorial process, related to tumor characteristics, clinical staging, the disease’s aggressiveness, tumor sites, and additional factors caused by disease progression. New biomarkers have been under investigation over the years in the attempt to correlate them to the risk of thrombosis, aiming to develop interventions that improve the clinical evolution of these cancer patients.

## INTRODUCTION

Cancers and their various treatments are recognized as independent risk factors for development of venous thromboembolism (VTE).^1^^,^ 2 The clinical association between neoplasms and hypercoagulability has been known for more than a century and thromboembolic events are more frequent in cancer patients – one in five cancer patients will develop VTE during the natural course of the disease.^3^

Venous thromboembolism encompasses a spectrum of clinical presentations that range from deep venous thrombosis (DVT) and superficial venous thrombosis to pulmonary embolism (PE).^4^^,^ 5 It is the second most common cause of death among patients with neoplasms, among whom one in seven deaths is related to complications, especially when in hospital treatment. Sixty percent of these patients have cancer in a single site or limited metastatic disease. According to Prandoni et al., they might survive for longer if they did not develop DVT or PE.^6^

The most prevalent types of cancer among patients with VTE are breast, colorectal, and lung cancer, which reflects the prevalence of these neoplasms in the population in general.^2^^,^ 7 ^-^ 9 Although solid tumors have historically been more associated with VTE, more recent data suggest that the level of risk is similar in patients with cancers of hematological origin.^10^^-^ 12

There are several different mechanisms that overlap and interact and can explain the increased incidence of VTE among cancer patients. In 1865, Trousseau observed that some patients had unexpected thrombotic events that were uncommon, with a migratory pattern, and then later manifested a visceral malignancy.^13^^,^ 14 The Trousseau syndrome, as it is known, can be described in many ways, including spontaneous VTE in association with a silent neoplastic disease, which in some cases may be the first manifestation of cancer.^4^^,^ 5 ^,^ 15

This syndrome has been widely used to encompass all aspects of cancer-related VTE. However, the risk of VTE is not equal for all patients with cancer or for the same patient over time.^1^^,^ 12 ^,^ 16 Certain factors, such as the characteristics of tumors, anatomic site, degree of aggression, and the patient’s clinical conditions make the development of VTE at a given point in the course of the disease more or less likely.^4^^,^ 17 ^,^ 18

## EPIDEMIOLOGY

There are many ongoing studies designed to help define with greater precision the prevalence of cancer-related VTE, as it is believed that the association is still underestimated. Studies have shown that many patients who had developed VTE were then diagnosed with some type of cancer in the 12 months following the thromboembolic event.^2^^,^ 3 ^,^ 9 The magnitude of this complication is such that it is estimated that cancer patients who develop VTE have a 94% probability of death in the 6 months following the episode. Therefore, VTE can be considered a negative predictive marker of survival in cancer patients.^18^^-^ 21

Cancer is itself associated with a four times greater risk of development of VTE, while chemotherapy increases the risk sixfold. Patients on cytotoxic treatment account for 13% of VTE episodes in the oncological population.^6^^,^ 8 ^,^ 22 ^,^ 23 A retrospective cohort study described by Blom et al. in 2005 found that patients on chemotherapy were at 2.2 times greater risk of developing VTE.^1^^,^ 11 ^,^ 24 The incidence of postoperative VTE in cancer patients is twice that of postoperative VTE in patients free from neoplasms.^6^^,^ 9 ^,^ 10 Factors such as prolonged immobilization and placement of central venous catheters also increase the risk of VTE in this group.^10^^,^ 25 As a result, the approximate annual incidence of VTE in a population with cancer can be as high as 1/200 patients.^26^ However, to a great extent, the incidence rates of VTE in patients with different types of neoplasms remain unknown because of the heterogeneous nature of the population and the difficulties involved in conducting large scale epidemiological studies.

Cancer patients are also at an elevated risk of VTE recurrence, particularly during the months following withdrawal of anticoagulant treatment. This risk can be as much as 2 to 3.5 times greater than for patients who develop VTE unrelated to cancer.^15^^,^ 21 ^,^ 27 During follow-up of patients, it was observed that a recurrence of VTE can occur even when they are on full anticoagulation, which suggests that the disease is more aggressive and prognosis is worse.^1^^,^ 6 ^,^ 27

## RISK FACTORS AND PATHOGENIC MECHANISM OF THROMBI FORMATION

Assessment of the risk of VTE is a dynamic process that involves a series of factors such as advanced age, sex, ethnicity (risk is higher in African Americans and lower among Asians), tumor sites (brain, pancreas, stomach, lung, bladder, gynecological tumors, or hematological origin), disease stage, and initial period after diagnosis.^15^^,^ 28 ^-^ 30 There are also factors related to treatment, such as surgery, hospital admissions, chemotherapy, antiangiogenic therapies, erythropoiesis-stimulating agents, and high pre-chemotherapy platelet counts.^10^^,^ 12 ^,^ 31 ^,^ 32

The pro-thrombotic properties specific to each type of tumor contribute to the process of tumoral growth and dissemination. Neoplastic cells can activate coagulation mechanisms by means of many different substances, procoagulants, fibrinolysis inhibitors, cytokines, cysteine protease, proinflammatories and pro-angiogenics, and by direct interaction with vascular endothelium, leukocytes, and platelets.^33^^-^ 36

Thrombin is the enzyme that ultimately effects the mechanisms of coagulation and both its formation and production of fibrin, the final product of activation of blood coagulation, are dependent on the mechanisms of tumor progression. Additionally, pro-thrombotic tumor properties can interfere in malignancy through independent coagulation mechanisms. Some of the most important mediators and mechanisms of development of cancer-related VTE will be described below.

## VIRCHOW’S TRIAD

First described in 1856 by Rudolf Virchow, and currently defined as venous stasis, endothelial injury, and hypercoagulability, the three key elements of Virchow’s triad, combined with current information about elements in the blood and their complex interactions in the pathophysiologic process of thrombogenesis, are useful tools for explaining the etiopathogenesis of VTE in cancer patients.^37^ Cancer patients can exhibit abnormalities of the vascular endothelium caused by the disease itself and/or secondary to the treatments they undergo. Additionally, they are also subjected to prolonged immobilizations during the course of the disease and its treatment and they undergo hematological changes caused by tumor activity.

**Venous stasis:** prolonged bed rest and extrinsic compression of blood vessels by tumoral masses can cause venous stasis.

**Endothelial injury:** is secondary to many factors that act locally, such as direct invasion of veins by tumors or by fitting central venous catheters, or remotely, such as endothelial injury secondary to chemotherapy treatment.^38^ The endothelium produces substances such as nitric oxide and prostacyclin, which are vasodilators and maintain platelets in the unactivated state, preventing them from aggregating. However, when the endothelial layer is ruptured, platelets are exposed to subendothelial ligands, for which they have specific receptors that initiate their activation process.^38^ By releasing mediators, the inflammatory process stimulates the endothelium to produce thromboplastin (TP) and type I plasminogen activator inhibitor (PAI-1), and to reduce synthesis of thrombomodulin, thereby decreasing its protective capacity.^39^ This mechanism promotes fibrin production through upregulation of TP and production of microparticles. Patients with neoplasms have elevated TP levels in circulation.^36^^,^ 39 In addition to TP, procoagulatory factors of neoplasms include production of thrombogenic substances such as coagulation factor and inflammatory cytokines, and tumor cells also interact with monocytes, macrophages, platelets, and endothelial cells.^36^^,^ 39

**Hypercoagulability:** in cancer patients, hypercoagulability is generated by a complex combination of mechanisms^36^:

- Release of microparticles derived from the tumor, rich in powerful procoagulatory tissue factors and cytokines capable of causing endothelial activation.
- Damage to the defense mechanisms of endothelial cells.
- Reduction in the plasma levels of the natural coagulation inhibitors antithrombin and proteins C and S.
- Increased adhesive interactions between tumor cells, vascular endothelial cells, platelets, and monocytes/macrophages, mediated by selectin interactions.^36^^,^ 37 ^,^ 40

It is presumed that activation of coagulation in cancer patients is simply a host reaction to development of the tumor. It would not therefore play a fundamental role in the molecular events that lead to development of the cancer.^36^^,^ 40

## PROCOAGULATORY SUBSTANCES

Tumor cells produce procoagulatory substances such as TP, tumor necrosis factor (TNF), and vascular endothelial growth factor (VEGF), which are involved in growth of the tumor mass and in activation of coagulation mechanisms.^35^ Thromboplastin is the primary activator of coagulation in healthy people. It is expressed on the surface of the majority of non-vascular cells and forms a complex with factor VII (FVII) to activate factors IX (FIX) and X (FX) by proteolysis.

In many diseases, including cancer, TP circulates in higher quantities in the form of microparticles. It is of interest to note that TP appears to be predictive of tumor aggression in humans and has been correlated, although retrospectively, with increased tumor angiogenesis, rapid growth rate, metastases and, finally, with propensity to develop VTE.^36^

Expression of TP is rigidly controlled in normal vascular cells. However, it appears that expression of TP is increased by neoplastic cells, induced by inflammatory stimuli, such as the cytokines interleukin 1 and TNF, and also by bacterial lipopolysaccharides. Therefore, coagulation of blood by TP can be caused directly by its expression on the surface of neoplastic cells or indirectly by its action in endothelial cells, monocytes, macrophages, and fibroblasts, after inflammatory stimulation.^4^^,^ 24

Regulation of expression of TP in tumor cells is controlled, on the molecular level, by several oncogenes, as appears to be the case of cyclooxygenase 2 (COX-2), an important regulator of platelet function, and of PAI-1, a fibrinolysis inhibitor. Additionally, it has been demonstrated that binding of protease-activated receptors (PARs) by TP, FVIIa, FXa, and/or thrombin is important to tumor angiogenesis, growth, and metastasis.^40^^-^ 43

With regard to the emerging role of non-coagulatory TP activity, of particular relevance is its capacity to modulate expression of VEGF by neoplastic cells and normal vascular cells. This property regulates tumor neovascularization and provides an important link between cancer patients and activation of coagulation, inflammation, thrombosis, tumor growth, and metastasis.^42^^,^ 44

The coagulation proteins in the blood perform at least two important roles in tumor biology: the intravascular and extravascular procoagulatory role, which leads to deposition of fibrin; and improvement of tumor cells in angiogenesis, growth, and metastasis. This double function occurs for TP and for the FVII and thrombin complex, which bind to one another and activate PARs in tumor cells, endothelial cells, and platelets.^40^^,^ 42 The resulting thrombotic manifestations are intimately related to tumor biology and make the patient susceptible to development of VTE because of the stimulation caused by tumor growth and the pathophysiologic mechanisms involved in tumor genesis.

It has also been suggested that in some patients with cancer, the tumor generates cysteine protease, thereby initiating blood coagulation, as was shown in a 1981 study by Gordon et al.^45^ They described cysteine protease, which directly activates FX in the absence of FVII. The current consensus is that this protease may play an important role in the prothrombotic state of some neoplasms, but data to prove this are still lacking.^36^^,^ 40

## P-SELECTIN

P-selectin is an adhesion molecule that interacts with platelets, endothelial cells and leukocytes. It increases TP expression in endothelial cells and monocytes, and elevated plasma levels have been associated with an increased risk of VTE in cancer patients. This can be used to discriminate between different levels of VTE risk^45^^,^ 46; but its use as a marker of risk is currently limited by the lack of availability of wide spectrum tests in clinical practice.^3^^,^ 10 ^,^ 45 ^,^ 46

## BIOMARKERS FOR RISK OF VTE

In addition to TP from tumor cells, circulating TP and soluble P-selectin, mentioned above, there are other possible biomarkers for the risk of VTE, such as high platelet count, elevated pre-chemotherapy white blood cell count, D-dimer and C-reactive protein.^10^^,^ 12 ^,^ 47

Leukocytosis (> 11,000/mm^3^) has recently been identified as an independent risk factor for VTE, associated with increased risk in cancer patients at the start of chemotherapy. Additionally, the VTE rate in patients who had persistent leukocytosis after the first chemotherapy cycle was significantly higher than the rate among those with leukopenia. Leukocytosis may be a marker of greater cancer aggression that is not used in traditional prognosis indicators such as disease staging. An elevated pre-chemotherapy platelet count has also been identified as a risk factor for cancer-related thrombosis.^16^ Hemoglobin level (< 10 g/dl-1) is also considered a biological marker of thrombotic risk.^10^^,^ 12 ^,^ 36 ^,^ 48

Thrombocytosis defined as a platelet count greater than or equal to 350,000/mm^3^ was observed in 21.9% of 4,405 patients analyzed in a prospective study at the start of ambulatory chemotherapy treatment. These patients went on to exhibit a threefold greater VTE rate. The elevated risk of developing VTE associated with high platelet counts persists while the patient is on chemotherapy.^24^

## CONCLUSIONS

Activation of blood coagulation in patients with cancer is complex and multifactorial, which makes these patients especially susceptible to VTE. Neoplastic cells can activate the coagulation mechanism by means of several substances, such as cytokines, cysteine protease, and procoagulants and proinflammatories, in addition to through direct interaction with vascular endothelium, leukocytes, and platelets. The pro-thrombotic mechanisms are related to the patient and to the tumor type, which exert a specific action in the thrombotic process. It is therefore of fundamental importance to be aware of these mechanisms in order to remain alert to the possibility of VTE in cancer patients. As it is a frequent event, and with a negative impact on clinical evolution, once we identify the subgroup most likely to develop VTE, we will be able to intervene more rapidly either with prophylaxis or with the most effective treatment for this population, thus leading to lower morbidity rates and longer survival times.
